# Immune and Inflammatory Cells in Thyroid Cancer Microenvironment

**DOI:** 10.3390/ijms20184413

**Published:** 2019-09-07

**Authors:** Silvia Martina Ferrari, Poupak Fallahi, Maria Rosaria Galdiero, Ilaria Ruffilli, Giusy Elia, Francesca Ragusa, Sabrina Rosaria Paparo, Armando Patrizio, Valeria Mazzi, Gilda Varricchi, Gianni Marone, Alessandro Antonelli

**Affiliations:** 1 Department of Clinical and Experimental Medicine, University of Pisa, 56126 Pisa, Italy; sm.ferrari@int.med.unipi.it (S.M.F.); ilaria.ruffilli@gmail.com (I.R.); e.giusy_87@hotmail.it (G.E.); francescaragusa86@gmail.com (F.R.); sabrinapaparo@gmail.com (S.R.P.); armandopatrizio125@gmail.com (A.P.); mazzivaleria@gmail.com (V.M.); 2 Department of Translational Research and of New Technologies in Medicine and Surgery, University of Pisa, 56126 Pisa, Italy; poupak.fallahi@unipi.it; 3 Department of Translational Medical Sciences and Center for Basic and Clinical Immunology Research (CISI), University of Naples Federico II, 80138 Naples, Italy; mrgaldiero@libero.it (M.R.G.); gildanet@gmail.com (G.V.); marone@unina.it (G.M.); 4 WAO Center of Excellence, 80138 Naples, Italy; 5 Institute of Experimental Endocrinology and Oncology “Gaetano Salvatore” (IEOS), National Research Council (CNR), 80131 Naples, Italy

**Keywords:** immune cells, immune checkpoints, macrophages, mast cells, neutrophils, tumor microenvironment, anaplastic thyroid cancer, papillary thyroid cancer, differentiated thyroid cancer, poorly differentiated thyroid cancer

## Abstract

A hallmark of cancer is the ability of tumor cells to avoid immune destruction. Activated immune cells in tumor microenvironment (TME) secrete proinflammatory cytokines and chemokines which foster the proliferation of tumor cells. Specific antigens expressed by cancer cells are recognized by the main actors of immune response that are involved in their elimination (immunosurveillance). By the recruitment of immunosuppressive cells, decreasing the tumor immunogenicity, or through other immunosuppressive mechanisms, tumors can impair the host immune cells within the TME and escape their surveillance. Within the TME, cells of the innate (e.g., macrophages, mast cells, neutrophils) and the adaptive (e.g., lymphocytes) immune responses are interconnected with epithelial cancer cells, fibroblasts, and endothelial cells via cytokines, chemokines, and adipocytokines. The molecular pattern of cytokines and chemokines has a key role and could explain the involvement of the immune system in tumor initiation and progression. Thyroid cancer-related inflammation is an important target for diagnostic procedures and novel therapeutic strategies. Anticancer immunotherapy, especially immune checkpoint inhibitors, unleashes the immune system and activates cytotoxic lymphocytes to kill cancer cells. A better knowledge of the molecular and immunological characteristics of TME will allow novel and more effective immunotherapeutic strategies in advanced thyroid cancer.

## 1. Introduction

The characteristics that a normal cell must obtain, to develop malignancy, are the intrinsic ones of the tumor cell (e.g., cell-autonomous growth and resistance to apoptosis) and the susceptibility to an inflammatory tumor microenvironment (TME) [1,2]. In addition, cancer cells can evade the anticancer immune response and proceed to form tumors [1,2,3].

Cancer immune surveillance is an important host protection process to inhibit tumorigenesis and to maintain cellular homeostasis. There are essentially three phases: elimination, during which newly transformed cells can be identified and destroyed by natural killer (NK) cells, cytotoxic CD8^+^ lymphocytes, and other immune cells; equilibrium, in which the immune system exerts a selective pressure and the most immunogenic cancer cell clones are eliminated; and escape, in which cancer cell clones resistant to immune attacks survive and proliferate to form tumors [4]. Therefore, the immune system plays a central role in tumorigenesis and tumoral cells are recognized and eliminated through different immune mechanisms.

To achieve tumor recognition and control, cancer cells are identified and killed by NK cells, then the tumor cell fragments are phagocytized by macrophages. Neoantigens released by cancer cells are processed by dendritic cells (DCs), which in turn secrete inflammatory cytokines and present tumor neoantigens to T cells. The subsequent activation of T and B cells fosters the expansion of tumor-specific T cells and the production of antibodies against neoantigens [5].

By the recruitment of immunosuppressive cells, decreasing the tumor immunogenicity, or through other immunosuppressive mechanisms, tumors can impair the host immune cells in TME and escape their surveillance [6]. Many tumors show the presence of immune infiltrating cells, similar to those recruited during the resolution phase of wound healing [7], causing a block of cytotoxic T lymphocyte- or NK-mediated killing of transformed cells [8]. Moreover, the presence of myeloid-derived suppressor cells (MDSCs), tumor-associated macrophages (TAMs) polarized toward an M2 phenotype, and tumor-associated mast cells (TAMCs) can help the tumor cells to escape from immune killing [9,10,11].

Furthermore, regulatory T cells (Tregs) (CD4+/CD25+ T lymphocytes) are elevated in primary and metastatic tumors and they are associated with poor prognosis [12]. These cells exert their immunosuppressive action producing cytokines (i.e., interleukin (IL)-10, transforming growth factor (TGF)-β, and IL-35) or inhibiting the activation of the T-cell receptor (TCR) [13]. For these reasons, Tregs are a potential therapeutic target which can improve tumor immunity and eventually provide clinical benefit [14].

Cancer cells can decrease their antigenicity to escape their elimination exerted by tumor-specific T cells. Tumor cells can downregulate the expression of tumor antigens [15] or decrease their ability to present antigens through the loss of major histocompatibility complex (MHC) I expression. Otherwise, tumors, if antigenicity for immune recognition is maintained, survive to tumor-specific T cells with the upregulation of soluble or membrane-associated immune-inhibitory molecules [16].

Among the membrane-associated molecules, the cytotoxic T-lymphocyte antigen 4 (CTLA-4), programmed cell death protein-1 (PD-1), and programmed cell death ligand-1 (PD-L1) and 2 (PD-L2), also known as “immune checkpoints”, are the principal inhibitory molecules to maintain the physiologic self-tolerance. During tumorigenesis, the overexpression of immune checkpoints inhibits several aspects of the anti-tumor immune response [17].

Among the soluble factors, the production of metabolic enzymes such as arginase (ARG) and indoleamine 2,3-dioxygenase 1 (IDO1) or of immunosuppressive cytokines (for example, TGF-β and IL-10) can contribute to the anticancer immune response suppression [18,19,20].

## 2. Immunological Cells in Thyroid Cancers (TCs) Microenvironment

**Figure 1:**
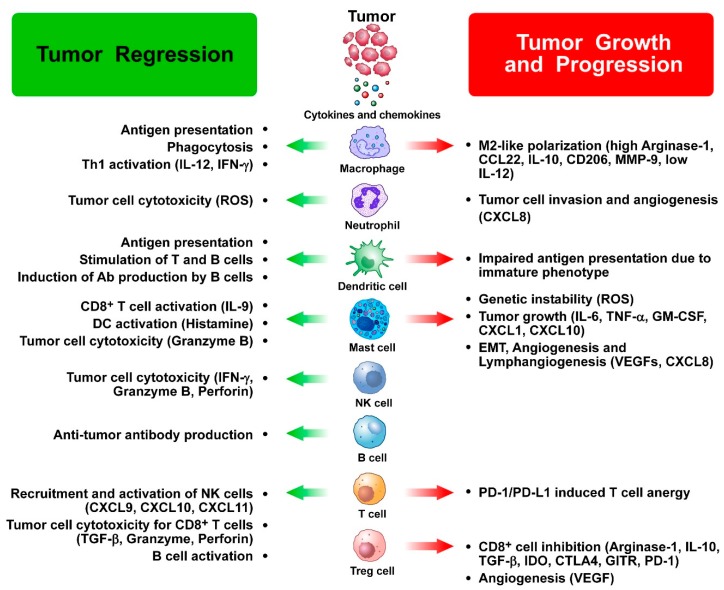
Dual role of immune cells in thyroid cancer growth and progression. Tumor-infiltrating immune cells can exert both antitumor and protumor functions in thyroid cancer and interact with each other as well as with cancer cells. A number of soluble factors (cytokines, chemokines, angiogenic and lymphangiogenic factors) released by immune cells mediate the protumor as well as the antitumor effects of immune cells in thyroid cancer (see text for details). List of abbreviations: Ab: antibodies; CTLA-4: Cytotoxic T-lymphocyte antigen 4; DC: dendritic cell; EMT: epithelial-to-mesenchymal transition; GITR: glucocorticoid-induced tumor necrosis factor family receptor; IDO: indoleamine 2,3-dioxygenase; IFN: interferon; IL: interleukin; MMP: matrix metalloprotease; NK: natural killer; PD-1: programmed cell death-1; PDL-1: programmed death-ligand 1; ROS: reactive oxygen species; TGF: tumor growth factor; TNF: tumor necrosis factor; VEGF: vascular endothelial growth factor.

A correlation has been shown between positive or negative clinical outcomes in TC patients and the density and types of tumor-infiltrating leukocytes [21,22]. In fact, different types of leukocytes have distinct roles in TCs before therapeutic interventions [23] (Figure 1).

### 2.1. Myeloid Cells

Myeloid-derived suppressor cells (MDSCs) are phenotypically heterogeneous, not fully mature, myeloid cells [24]. MDSCs are rare in healthy subjects but are elevated in cancer patients, where they show a strong immunosuppressive potential [25] and are associated with a poor prognosis. Elevated preoperative levels of circulating MDSCs have been reported in TC patients in comparison to benign nodules or other noncancerous thyroidal diseases [26,27]. Moreover, a correlation has been found between the number of circulating MDSCs and aggressiveness of differentiated TC (DTC) [27]. In TCs patients, the prognosis could potentially be ameliorated with the differentiation of MDSCs into mature myeloid cells or through their depletion or functional inhibition by different treatments, such as nitric oxide inhibitors, chemotherapy, tyrosine kinase inhibitors (TKIs), and/or bisphosphonates [28]. In particular, the antitumor activity of the TKI sunitinib has been demonstrated in advanced differentiated TC [29]. Sunitinib seems to decrease MDSCs that correlates with increased tumor-specific responses produced by cancer vaccines in preclinical models [30].

The density of tumor-associated macrophages (TAMs) changes in the distinct subtypes of TCs [31]. In particular, anaplastic thyroid cancers (ATCs) have the highest density of TAMs in TME, and this correlates with poorer prognosis [31]. In papillary thyroid cancers (PTCs), the presence of TAMs is lower but a comparable correlation exists with clinical outcomes, as larger tumors, more lymph node metastases, and decreased survival [31,32,33,34]. In vitro studies reported that TAMs can promote invasiveness of human TC cell lines through CXCL8/IL-8 secretion [34], and this was confirmed by enhanced spreading of human PTC cells after treating immunodeficient mice with CXCL8/IL-8 [34]. On the contrary, a retrospective study reported a positive association between the number of tumor-infiltrating macrophages and enhanced disease-free survival in TCs patients [35]. Further studies will be necessary to clarify these contradictory results. In particular, it could be important to distinguish between inflammatory type 1 and suppressive type 2 TAMs, as the first ones are usually associated with better outcomes in other types of cancer [36,37].

DCs play a central role in presenting antigens and in regulating immune functions through the secretion of cytokines. For these reasons, they play key roles in the induction of immunity. DCs are infrequently found in the thyroid, but they are increased in human PTCs [38]. Tumor-infiltrating DCs often display an immature phenotype, expressing low levels of co-stimulatory molecules and high levels of regulatory molecules, leading to an altered antigen presentation [39,40]. Immature DCs (iDCs) poorly induce T cell and NK cell-mediated responses and they can even inhibit immune responses by producing suppressive cytokines (i.e., IL-10 and TGF-β) [41]. Tregs and DCs participate in the immunosuppressive conditioning of TME. In a model of pancreatic ductal adenocarcinoma, Tregs suppressed the function of tumor-infiltrating DCs, inhibiting the expression of co-stimulatory ligands and the activation of CD8^+^ T cells [42]. Tregs and DCs are elevated in human PTCs [43]; therefore, the interruption of Tregs and DCs interactions in TCs could be a good therapeutic strategy. The function of tumor-infiltrating DCs can be restored by blocking immunosuppressive pathways, such as those associated with PD-1, secretion of IL-10, and production of lactic acid [40,44].

### 2.2. Neutrophils

Neutrophils are involved in the acute phase of the inflammatory response and represent the first line of defense against extracellular microbes [45]. Quite recently, new roles have been reported for neutrophils in immune and inflammatory responses [46,47,48].

The density of tumor-associated neutrophils (TANs) in human cancer samples correlated with a better patients’ clinical outcome [49,50,51,52,53,54,55], even though their functional roles are still controversial. On the one hand, TANs can favor genetic instability of cancer cells and release cytokines (such as onconstatin-M, VEGF-A) or granule proteins (such as neutrophil elastase) involved in the promotion of cancer cell proliferation, invasiveness, and angiogenesis [56,57,58,59,60]. On the other hand, antitumor functions have been described for neutrophils, i.e., able to kill tumor cells, stimulate the T- cell-dependent anti-tumor response, and inhibit angiogenesis [55,61,62,63].

The prognosis of TC patients is still difficult to define, owing to the heterogeneity of the disease [64]. The “neutrophil-to-lymphocyte ratio” (NLR) was associated with tumor progression [65] since an elevated NLR correlated with larger tumor volume and a higher risk of recurrence.

Lee et al. evaluated 151 TC patients, reporting a significant decrease in NLR after treatment in those with low risk of recurrence, those with stage I disease, and those with an excellent response to therapy [66]. At follow-up, NLR significantly increased (*p* = 0.012) in patients with a structural incomplete response. On multivariate analysis, incomplete response to therapy was associated with an increased NLR (OR = 13.68). The authors concluded that an increase in systemic inflammation after treatment (measured by NLR) is independently associated with an incomplete response to therapy in DTC [66]. However, NLR does not allow to discriminate malignant from benign lesions [67]. Furthermore, NLR does not correlate with the risk of occult metastasis or with patients’ survival [68].

The presence of infiltrating neutrophils in human TC and the phenotypic and functional characteristics of “tumor-educated” neutrophils have been recently evaluated. Indeed, TC cells were able to recruit neutrophils through the release of CXCL8/IL-8 and to improve their survival through the release of granulocyte colony-stimulating factor (GM-CSF). TC cells upregulated neutrophils’ proinflammatory activities and the expression of factors able to promote tumor progression. Moreover, in human TC samples, neutrophil density correlated with tumor size, suggesting a potential tumor-promoting role of TANs in TC [69].

### 2.3. NK Cells

NK cells play a central role in cancer immunosurveillance through killing cancer cells [70,71]. However, few solid tumors respond to NK cell-mediated immunotherapy owing to the resistance to the lysis induced by NK cells and the reduced homing and infiltration of NK cells into tumors [72]. ATC cell lines in vitro are responsive to NK cell-mediated lysis, leading to hypothesize that TC can take advantage of immunotherapies that incorporate in TME the recruitment of activated NK cells [72]. Furthermore, the cells secreted CXCL10/IP-10 after the stimulation with interferon (IFN)-γ [73] and showed the capability to attract CXCR3^+^ NK cells [72]. The transfer of ex vivo-expanded NK cells to in vivo-animal model of ATC with the appropriate cellular environment could represent a promising therapeutic model.

Tumor immunosuppression is an obstacle to effective immunotherapy with NK cells. Intratumoral NK cells have an inactive phenotype when compared to blood NK cells. When NK cells are cocultured with ATC, which expresses elevated levels of COX2, the NKG2D (the activation receptor for NK cells that increases the lysis of tumoral cells) was downregulated, when compared to those cocultured with COX2-negative cell lines [72]. The administration of neutralizing antibodies to prostaglandin E2 (PGE_2_) could rescue this downregulation, suggesting that this eicosanoid downregulates NK cell activity. Other studies reported NK dysfunction in tumor-bearing mice. A diminished splenocyte mediated cytotoxicity in thyroid tumor-bearing LSL-BrafV600E/TPO-Cre mice (that express mutant BrafV600E transcripts under the endogenous Braf promoter between 3 and 10 days postnatally and spontaneous PTC developed at about the age of 5 weeks [74]) with respect to normal LSL-BrafWT/TPO-Cre mice was shown [75]. NK and CD8^+^ T cells mediated this cytotoxicity and the treatment with exogenous IL-12 and anti-TGF-β partially restored this diminished cytotoxicity [75].

Additional studies are necessary to clarify the role of NK cell dysfunction in TC to obtain effective therapeutic strategies.

### 2.4. T Cells

Different types of cancers, such as metastatic melanomas [76], ovarian [77,78], colorectal [79,80], and breast cancers [81], show a good outcome in the presence of lymphocytic infiltration. In human PTC, the density of lymphocytes is correlated with improved overall survival and lower recurrences [82,83]. Another study showed that proliferating lymphocytes (identified for the ability to express the nuclear antigen Ki-67) could predict the enhanced disease-free survival in children and young adults [84]. Infiltration of CD8^+^ T cells in TCs was associated with enhanced disease-free survival [6,35]. CD8^+^, CD4^+^ T cells, and B cells were positively correlated with reduced tumor sizes [35]. On the contrary, another study found a higher risk of relapse in the presence of elevated infiltration of CD8^+^ T cells [85]. IL-2 and IL-15 regulate the expression of the cytolytic proteins granzyme and perforin [86,87]. For this reason, a treatment inducing the overexpression of IL-2/IL-15 in TC TME could permit to activate T cells with cytotoxic activity. Even if the systemic delivery of IL-2 can be toxic, new manners to get IL-2 into a tumor have been evaluated (i.e., IL-2 encoded by an oncolytic virus or linked to a tumor-associated ligand) [88,89].

Tumor-infiltrating T lymphocyte activity can be impaired by the cancer microenvironment. Inhibitory receptors, such as PD-1, are immune checkpoints, able to limit T cell responses, inducing anergy or apoptosis [90]. The physiological role of immune checkpoints is to downregulate the immunologic response after the initial induction of a protective response and to avoid the risk of autoimmunity. In tumors, autoreactive T cells specific for “cancerous self” are needed to eliminate cancer cells. Inhibitory receptors as PD-1 are upregulated on T cells in the same manner as the expression of elevated levels of cognate ligands (i.e., PD-L1 and PD-L2), leading to diminished production of IFN-γ and the cytotoxic potential [91]. Of note, the expression of PD-1 seems to be associated with the presence of TC-infiltrating CD8^+^ and CD4^+^ T cells [92], suggesting that immune checkpoint inhibitors (ICIs) treatment could reverse the cytotoxic T cell responses in TCs.

Tregs switch off immune responses, favoring disease progression and metastases to lymph nodes in different tumors [93]; their presence in PTCs is associated with more aggressive disease [94]. A paper evaluated the clinicopathologic significance and roles of Treg in PTC patients with/without Hashimoto’s thyroiditis (HT) [95]. The percentage of CD4+CD25+CD127low/- Treg among CD4+ T cells was significantly more elevated in PTC patients than in multinodular goiter (MNG) patients. A higher number of tumor-infiltrating FoxP3+ Treg in primary PTC and metastatic lymph nodes tissues was present, and no FoxP3 expression in the MNG tissues was found. In peripheral blood and tumor tissues, a higher percentage of Treg was associated with extrathyroidal extension and lymph nodes metastasis. The percentage of CD4+CD25+CD127low/- Treg among CD4+ T cells in peripheral blood of PTC patients with HT was significantly lower, while the infiltration of FoxP3+ Treg in tissues of PTC with HT was increased. The authors concluded that the percentage of Treg increased in peripheral blood and in the tumor tissues of PTC patients in comparison to that of MNG patients, and it was associated with aggressiveness [95].

T helper 17 (Th17) cells and follicular helper T (Tfh) cells are regulatory subsets of CD4^+^ T lymphocytes [96], but their roles in TCs have not been studied exhaustively. Few data are present about the prognostic value of CD4^+^ T cells in TCs, even if it is possible that the evaluation of the CD8^+^ cytotoxic-regulatory T cell ratio in TCs might be important, as it has been shown in other tumors [21].

A higher density of T lymphocytes that did not express CD4 or CD8 has been reported in TC compared to patients with autoimmune thyroid diseases [97]. These intratumoral double-negative T cells seem to decrease the growth and cytokine production of neighboring activated effector T cells [92]. For this reason, reducing the number of these cells in TCs could help immune-mediated therapies.

### 2.5. Mast Cells (MCs)

Mast cells are tissue-resident immune cells which have a widespread distribution in nearly all tissues and human cancers [98,99]. These cells are in close proximity to epithelia, fibroblasts, and blood and lymphatic vessels, and are involved in wound healing, angiogenesis, lymphangiogenesis and tumor growth [100,101,102]. MCs are recruited into TME by several tumor-derived chemotactic factors such as stem cell factor (SCF), vascular endothelial growth factors (VEGFs), chemokines, and cytokines [103]. Moreover, tumor-associated mast cells (TAMCs) can be activated by several factors within TME such as hypoxia, adenosine, PGE2, chemokines, and immunoglobulin free light chains [103,104]. MCs play a protumorigenic role in the majority of solid and hematologic tumors, but their contribution to cancer varies according to stage of tumorigenesis [105,106,107,108] and to their microlocalization in tumors [109,110,111].

#### 2.5.1. MCs in TC

Only few papers evaluated the relationship between MCs and TC [112]. MCs infiltration was reported in 95% of PTC samples in which the extent was associated with extrathyroidal extension of tumors, while normal thyroid tissues stained negative for tryptase, a particular MCs marker. The presence of MCs was evaluated also in poorly differentiated thyroid cancer (PDTCs) and ATCs by immunohistochemistry (IHC), showing it both in PDTC and ATC, and their density correlates with tumor invasiveness [113] (Table 1).

A higher presence of MCs was shown in the intratumoral and peritumoral areas of follicular variant of PTC in comparison to adenoma [114]. Therefore, MCs density could help to discriminate between malignant and benign forms of follicular thyroid lesions [114].

In vitro studies in human MCs lines (HMC-1 and LAD2) and human primary MCs isolated from human lung (HLMC) reported that VEGF-A induced MCs chemotaxis, and it has been found that different TC cell lines release VEGF-A and other soluble factors activating MCs [112]. This activation was not mediated by IgE, but by mediators which are still unknown. Several mediators (i.e., IL-6, IL-1, TNF-α, histamine, and the chemokines IP-10, CXCL8/IL-8, and CXCL1/GRO-α) have been identified, analyzing MCs factors released after TC activation. TC cell proliferation, survival, and motility were stimulated by the mediators present in MCs-conditioned media. The binding of histamine to H1 and H2 receptors on PTC cells induced cell proliferation, even if with a lower effect than that induced by MCs-conditioned media. Interestingly, combining histamine with GRO-α and IP-10, an effect similar to that of MCs-conditioned media was exerted. These data were confirmed by immune-depletion experiments [112]. Importantly, the subcutaneous injection of MCs and TC cells in athymic mice expedited the growth of TC xenografts. TC cell xenografts recruited MCs injected in tumor site, and MCs injection induced an enhanced growth and vascularization of xenografts. Treating mice with sodium cromoglycate (cromolyn), a particular inhibitor of MCs degranulation, reduced these effects [112]. Collectively, these results indicate a protumorigenic role of MCs and their mediators in TC.

#### 2.5.2. MCs in Epithelial-To-Mesenchymal Transition (EMT) and Stemness

The raised motility and invasiveness of tumoral cells derive from the EMT activation, which is essential in tumor progression [115]. EMT is a genetic program, occurring during embryonic development or in response to injury, through which epithelial cells transdifferentiate and obtain a mesenchymal and invasive phenotype. Comparable signaling pathways, effectors, and regulators are present in pathological and physiological EMTs. EMT frequently occurs at the invasive front of various carcinomas [116] and can be provoked by cellular signals derived from tumor cells and TME.

Human TC cell lines, obtained from FTC, ATC, and PTC, undergo EMT once exposed to activated MCs-conditioned media and change morphology into a mesenchymal phenotype, they upregulate EMT markers and downregulate epithelial markers, and a functional EMT is activated [113]. Among the different mediators produced by MCs, TNF-α, IL-6, and CXCL8/IL-8 efficiently media-induced a functional EMT. Interestingly, only immunodepletion of CXCL8/IL-8, but not of IL-6 or TNF-α, blocked MCs-conditioned media-mediated EMT induction in TC cells. The addition of exogenous CXCL8/IL-8 reverted this effect, suggesting that this mediator plays a key role in EMT [113].

The connection between EMT and cancer stem cells (CSCs) has been evaluated in the distinct types of tumors [117]. EMT inducers or regulators could also induce cancer cells to acquire stem cell-like features, suggesting the existence of a cross-talk between EMT and the pathways that regulate stemness [117]. To isolate cells with stem-like characteristics, their capacity to grow in low adherence conditions, giving rise to cell spheroids, can be used [118,119,120]. MC-conditioned media or recombinant CXCL8/IL-8 treatment of TC cells led to the achievement of stemness characteristics more efficiently in comparison to unstimulated cells, suggesting that MC-conditioned media and CXCL8/IL-8 enhance TC cell stemness. Blocking CXCR1 and CXCR2, the CXCL8/IL-8 receptors, with neutralizing antibodies, a strong reduction of the ability of TC cells to form spheroid was shown [113]. CXCL8/IL-8 stimulated EMT/stemness of TC cells through the Akt-SLUG pathway [113]. When human PTC samples were analyzed by IHC with antibodies anti-tryptase and anti-OCT-4 (a stem cell marker), a positive correlation between MCs density (tryptase^+^ cells) and stemness features (OCT-4) was reported. The authors concluded that the release of certain mediators (i.e., CXCL8/IL-8) lead MCs to improve the acquisition of mesenchymal and stem-like characteristics of TC cells, therefore fostering cancer progression [113].

#### 2.5.3. Conclusive Remarks

**Figure 2:**
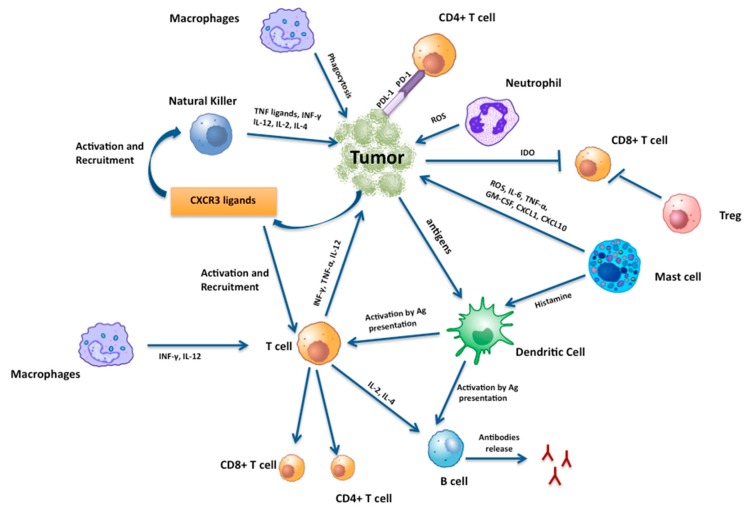
A schematic view of tumor-infiltrating immune cells interactions among each other and with the thyroid cancer cells.

The association between chronic inflammation and TC involves several components of the innate and adaptive immune system: (a) cells of the innate immune response (macrophages, mast cells, and neutrophils); (b) cells of the adaptive (lymphocytes) immune responses. These cells interact with tumor cells via chemokines, adipocytokines, and cytokines [121] (Figure 2).

## 3. Chronic Lymphocytic Thyroiditis (CLT) and TC

CLT is the most common autoimmune disorder reaching 10–20% prevalence in different populations, overall in females over 50 [122,123,124]. Several studies demonstrated the epidemiologic association, up to 38%, of CLT and TC (in particular PTC) [38,125,126]. In PTC, the lymphocytic infiltration seems to correlate with the severity of thyroiditis in normal tissues, indicating that immunologic mechanisms are involved in their pathogenesis [127]. In support of this hypothesis, the Warthin-like variant of PTC, constituted prevalently by papillae filled with a dense lympho-plasmacytic infiltrate and lined by oncocytic cells, is commonly associated with CLT. PTCs, with associated CLT, show a less extensive disease at diagnosis and improved disease-free survival [82,128], whereas patients with tumor-infiltrating lymphocytes in PTC, without CLT, had a higher disease stage and an elevated incidence of invasion and lymph node metastasis compared to patients without lymphocytes [94]. Different prospective study investigated the association between HT and thyroid malignancy, showing a higher rate of indeterminate cytology and a higher incidence of thyroid cancer in patients with HT [129,130].

Recently, it has been shown in vitro and in vivo that thyroid autoimmunity and TC (especially PTC) can be concomitant [131]. The exact mechanism at the basis of this association is unknown. Elevated thyroid-stimulating hormone (TSH) levels and thyroid autoimmunity were considered independent risk factors for TC; autoimmunity and inflammation, *per se*, are retained TC risk factors. Within TME, inflammatory cells, of both the innate (macrophages) and the adaptive (lymphocytes) immune responses, are interconnected with endothelial cells, adipocytes, fibroblasts, and extracellular matrix through adipocytokines, cytokines, and chemokines. Under the influence of transcriptional regulators (i.e., phosphoinositide-3 kinase/protein kinase-B, mitogen-activated protein kinases, or nuclear factor-kappa B), oncogenes connected to the distinct subtypes of TC promote their effect on TME [131].

## 4. The Role of Cytokines/Chemokines in TC

Chemokines are classified into four major subfamilies (CXC, CC, C, and CX3C) according to the presence of four conserved cysteine residues in the protein. By binding to specific G protein-coupled cell-surface receptors on target cells, they induce cell migration and activation. Continuous cell lines obtained from ATCs and well-differentiated thyroid carcinomas (WDTCs) secrete different cytokines and chemokines, such as GRO-α, GM-CSF, IL-1α, IL-6, CXCL8/IL-8, monocyte chemotactic protein-1 (MCP-1), and TNF-α [132,133,134,135,136,137,138,139].

In PTCs, the presence of oncoproteins, as rearrangements of the RET receptor (RET/PTC), RAS, and BRAF, induces a proinflammatory state upregulating several chemokines [132,133,134,135,136,137,138,139]. For instance, RET/PTC induces the expression of oncogenes involved in inflammation and tumor invasion, such as chemokines (CCL2, CCL20, CXCL8/IL-8, and CXCL12), chemokine receptor CXCR4, and cytokines (IL-1B, colony-stimulating factor 1, and GM-CSF) [135,137]. Thyrocytes engineered to express RET/PTC3 produce elevated levels of MCP-1, GM-CSF, IP-10, and IL-6 [137].

**Figure 3:**
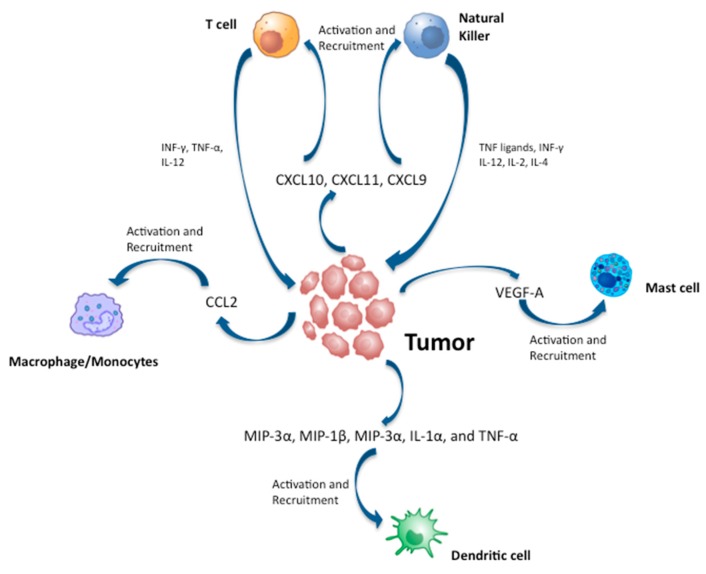
Cytokines and chemokines released in thyroid cancer microenvironment and their role in recruiting inflammatory cells.

It has been shown that the genes encoding chemokines CCL20, CXCL8/IL-8, and the adhesion molecule L-selectin are overexpressed in PTC in comparison to normal thyroid, independently from the RET/PTC or BRAF status, suggesting that these chemokines could be associated with tumor-related inflammation [133]. Approximately 95% of PTCs overexpress the Met protein, the receptor for hepatocyte growth factor (HGF) [132]. The HGF/Met interaction is biologically active in PTCs, causing the release of chemokines (i.e., macrophage inflammatory protein (MIP)-1α, MIP-1β, and MIP-3α) [132]. Among these chemokines/cytokines, VEGF-A attracts MCs [111], CCL2 attracts macrophages/monocytes [140], and MIP-3α, MIP-1β, MIP-3α, IL-1α, and TNF-α (markedly expressed in PTCs but slightly in FTCs) [141,142] attract DCs [132,133,135,143] (Figure 3). Interestingly in FTCs, which do not overexpress Met and other immune-related proteins and factors, the number of tumor-associated inflammatory cells is irrelevant [132].

## 5. The Role of TC Cells in Recruiting Inflammatory Cells in TC

In PTCs, RET/PTC rearrangements and activating mutations in the BRAF or RAS oncogenes activate a transcriptional program and lead to the upregulation of the IP-10 chemokine, which in turn stimulates proliferation and invasion [138]. Moreover, the presence of peroxisome proliferator-activated receptor (PPAR)-γ has been reported in thyroidal tissues, and PPAR-γ takes part in the modulation of inflammatory responses [144]. Treating normal thyroid follicular cells (TFC) with rosiglitazone (a PPAR-γ ligand), at near-therapeutical doses, inhibited IFN-γ-induced IP-10 secretion. These data indicated that PPAR-γ can be involved in the regulation of IFN-γ-stimulated chemokine expression in human thyroid autoimmunity [144].

PPAR-γ is considered a tumor suppressor gene, and the antiproliferative effects of the PPAR-γ ligands thiazolidinediones have been shown in human ATC and dedifferentiated PTC primary cell cultures [145].

In primary cultures of TFCs and PTCs, we reported that IP-10 was not released basally, but IFN-γ stimulated its secretion in a similar manner in both cell types, while TNF-α alone induced a slight but significant IP-10 secretion only in PTCs [146]. The cotreatment with IFN-γ and TNF-α had a synergistic effect on the IP-10 secretion from PTC cells, and to a lesser extent from TFC [146]. Moreover, thiazolidinediones had antiproliferative effects in PTC primary cells [146]. These data suggested dysregulation of IP-10 secretion in PTCs and the effects of thiazolidinediones on IP-10 were unrelated to the significant antiproliferative effect in PTC cells [146].

We evaluated also IP-10 levels in primary human ATC cell cultures and the effect of IFN-γ and/or TNF-α stimulation on its secretion [73,147]. Primary human ATC cells, but not primary TFC cells, spontaneously secreted IP-10. The treatment with IFN-γ induced the IP-10 secretion in a concentration-dependent manner, both in primary ATC and TFC cells, while TNF-α alone had no effect [73]. The cotreatment with IFN-γ and TNF-α induced a synergistic effect on the IP-10 secretion both in TFC and ATC cells, even if with a variable effect on the IP-10 release in different primary ATC cell preparations, while it was more reproducible in TFCs [73]. Moreover, primary ATC and TFC cells were treated with rosiglitazone in the presence of IFN-γ + TNF-α, and the effect on IP-10 secretion was inhibitory or stimulatory or nil in ATCs, and inhibitory in TFCs [73]. Rosiglitazone was able to reduce primary ATC cells proliferation. These results suggest that the pattern of modulation of IP-10 secretion by IFN-γ, TNF-α, or thiazolidinediones is extremely variable in ATC, indicating that the intracellular pathways involved in the chemokine modulation have different types of dysregulation [73].

We investigated also the pattern of secretion of CXCL9/MIG and CXCL11/ITAC in TFC and PTC primary cells in vitro [148]. MIG and ITAC were not secreted basally in both cell types, the treatment with IFN-γ induced the chemokines secretion, while TNF-α alone induced it only in PTC. Cotreatment with IFN-γ and TNF-α induced a synergistic effect on chemokines release from PTC and, to a lesser extent, from TFC cells. The treatment with PPAR-γ ligands in the presence of IFN-γ and TNF-α suppressed chemokines secretion in TFCs in a concentration-dependent manner, while stimulated it in primary PTC cells. PPAR-γ knocking down, by RNA interference technique in PTC cells, abolished the effect of PPAR-γ ligands on chemokines release. In PTC cells, PPAR-γ ligands reduced proliferation, and MIG or ITAC reduced significantly proliferation and migration. These results suggested to explore further the use of MIG or ITAC as antineoplastic agents in PTC [148].

The role of CCL2/MCP-1, the prototype Th2 chemokine, has been evaluated in primary human ATC cell cultures [149]. MCP-1 was secreted by tumor cells and exerted growth-promoting effects. Primary ATC cells released basally MCP-1 at a higher level than TFC cells. Among the 6 established ATC primary cell cultures, IFN-γ or TNF-α dose-dependently induced the MCP-1 secretion in 3/6 or 5/6, respectively, and in all TFC cells, while thiazolidinediones inhibited it in 3/6 ATC cells, and they had no effect in TFC cells. The treatment with pioglitazone, a PPAR-γ ligand, inhibited the proliferation of primary ATC cell cultures. We concluded that primary ATC cells release spontaneously MCP-1 and upon cytokine stimulation, with an extremely variable pattern of modulation, indicating a different type of dysregulation in the chemokine secretion. Additional studies are necessary to clarify whether MCP-1 could be considered as a biomarker in the follow-up of ATC patients [149]. On the whole, the above data underline the important role of TC cells in recruiting inflammatory cells into the TME.

## 6. The Inflammatory Role of Cancer-Associated Fibroblasts (CAFs) in Thyroid Cancer

CAFs surround the tumor cells and they participate in tumor initiation, tumor-stimulatory inflammation, metabolism, drug response, metastasis, and immune surveillance [150]. However, the role of CAFs in thyroid cancer is complex and somehow still contradictory.

A paper evaluated the association between expression of CAF-related proteins in PTC in relation to clinicopathologic factors in 339 PTCs [151]. It was shown that the expression of CAF-related proteins in stromal cells and cancer cells of PTC varied on the basis to histologic subtype, BrafV600E mutation, and subtype of stroma, and it was associated with shorter overall survival [151].

Furthermore, another paper studied the association between CAFs and cervical lymph node metastasis in PTC [152]. Among 78 PTC patients, 65 presented desmoplastic stromal reaction around the tumor. CAFs were found in 42 (64.6%) cases with desmoplastic stroma. At univariate analysis, it was shown that tumor size and CAFs were risk factors of lymph node metastasis. However, by a multivariate analysis, CAFs were the only independent risk factor of lymph node metastasis in these patients [152].

## 7. The Immune Landscape of TCs

Evaluating the specific patterns of immune cells infiltrating TCs, including not only their phenotypes but also the function, it is crucial to understand the immunological characteristics of different TCs. Tumors have been classified into 6 immune subtypes according to their transcriptomic and genomic data available in The Cancer Genome Atlas, through immunogenomics methods, that allowed to define the TME immune components. Through the use of this technique, PTCs have been subdivided in “inflammatory” tumors [153]. PTCs are cancer types with low mutational burden owing to low neoantigen expression that indicates a slight immunogenicity, but they have a substantial immune infiltrate accounting for the “inflammatory” immune subtype [17]. It is still unclear whether the PTC-associated inflammation depends on some intrinsic characteristics of the thyroid, as the presence and abundance of tissue-specific antigens, or on the frequent disruption of immunological tolerance and the subsequent propensity to autoimmunity. In any case, the presence of autoimmunity or of CLT has been associated with a good prognosis in TC and in other tumors [154,155]. In contrast, the presence of immunosuppressive cell populations has also been reported in PTCs and their density frequently correlated with a poor prognosis.

Moreover in TC, the immune subtype has been associated with specific genetic lesions and with the differentiation score. For example, elevated scores for DCs, macrophages, and MCs correlated with low thyroid differentiation score or with BrafV600E mutation in PTCs, and the expression levels of CTLA-4 and PD-L1 were more elevated in BrafV600E+ and in dedifferentiated TCs [156]. These data confirm what reported by earlier IHC studies showing that the BrafV600E mutation status was strictly associated with Tregs and immunosuppressive macrophage components, and with immunosuppressive markers, such as PD-L1 [157]. Furthermore, in PTCs, elevated expression levels of PD-L1 correlated with TAM and CD8^+^, CD4^+^, and Treg lymphocytic infiltrate [35,158]. PD-L1 positive expression in PTCs correlates with a higher risk of recurrence and reduced disease-free survival [159]. In PDTCs, the expression of PD-L1 was significantly associated with increased tumor size and multifocality. In metastatic PTCs, PD-1^+^ T-lymphocytes were present in lymph nodes, indicating their significant association with cancer lymph-nodal invasion and recurrent disease [160].

A recent paper presented data on the PD-L1 expression in 407 primary TCs with a median 13.7-year of follow-up, analyzing the associations between PD-L1 expression and clinicopathologic factors (such as TERT promoter, disease progression, and BRAF status). Tumoral PD-L1 was expressed in 6.1% of PTCs, 7.6% of follicular thyroid cancer (FTCs), and 22.2% of ATCs. The distribution of PD-L1 positivity was variable (*p* < 0.001) according to cancer histology types. The proportions of positivity in PD-L1 positive ATCs were more than 80%. PD-L1 in immune cells was positive in 28.5% of PTCs, 9.1% of FTCs, and 11.1% of ATCs. There was no significant association between PD-L1 expression and clinicopathologic variables, oncogenic mutation, disease progression [161].

Another paper evaluated PD-L1 expression levels in medullary thyroid cancer (MTC), demonstrating almost no expression of PD-L1 in MTC and accompanying inflammatory cells [162].

The expression of IDO1 in TCs sustained the immunosuppressive context and it was associated with a raised Treg infiltrate and with more aggressive clinicopathologic characteristics, such as extrathyroidal extension or multifocality [163,164].

## 8. Conclusions

There is overwhelming evidence that chronic smoldering inflammation has a protumorigenic role in TC [23,165,166]. The association between chronic inflammation and TC involves several components of the innate and adaptive immune systems, extracellular matrix, stroma, and adipose tissue [133]. Within the TME, cells of the innate (macrophages, mast cells, and neutrophils) and the adaptive (lymphocytes) immune responses communicated with fibroblasts, adipocytes, endothelial cells, and extracellular matrix via chemokines, adipocytokines, and cytokines [121].

In TCs, oncogenes promote proliferative effects on the TME, influenced by transcriptional regulators such as NF-kB, PI3K-AKT, and MAPK.

There is increasing evidence that cancer-related inflammation could be a useful target for novel diagnostic and therapeutic strategies in TC [167]. There is now evidence that different immune cells (e.g., macrophages, mast cells, neutrophils, lymphocytes) play a protumorigenic role, whereas other types play a protective role in tumorigenesis. Single-cell analysis of peritumoral and intratumoral immune cells in different types of TC could be of paramount importance to elucidate the functions of immune cells in TC TME.

Anticancer immunotherapy, especially ICIs, promote lymphocyte activation against cancer cells and inhibit immune-suppressive signals, leading to a sustained anti-tumor response [158]. Preclinical and preliminary clinical studies have reported promising results on the efficacy of monoclonal antibodies targeting PD-1/PD-L1 network in combination with BRAF inhibitors [168,169]. The results arising from several ongoing experimental and clinical studies will contribute to elaborate novel targeted immunotherapies for advanced TCs.

## Figures and Tables

**Table 1:** Immune cells in thyroid cancer microenvironment.

Immune Cells	Reported Data	References
**Tumor-associated macrophages**	ATCs have the highest density of TAMs in tumor microenvironment, and this correlates with poorer prognosis.	[31]
	In PTCs, the presence of TAMs is lower but a similar correlation exists with clinical outcomes, as more lymph node metastases, larger tumors, and decreased survival.	[31,32,33,34]
	In vitro studies reported that TAMs can promote invasiveness of human TC cell lines through CXCL8/IL-8 secretion.	[34]
	A retrospective study of TCs patients reported a positive association between the number of tumor-infiltrating macrophages and enhanced disease-free survival.	[35]
**Dendritic cells**	Immature DCs poorly induce T cell and NK cell-mediated responses and they can even inhibit immune responses producing suppressive cytokines, such as IL-10 and TGF-β.	[41]
	Tregs and DCs are elevated in human PTCs.	[43]
**Tumor-associated neutrophils**	An independent association is found between NLR increase and an incomplete response to therapy in DTC.	[66]
	In human TC samples, neutrophil density correlated with tumor size, suggesting a potential tumor-promoting role of TANs in TC.	[69]
**Natural killer cells**	ATC cell lines in vitro are responsive to NK cell-mediated lysis. Furthermore, the cells secreted CXCL10/IP-10 when stimulated by IFN-γ and demonstrated an ability to attract CXCR3+ NK cells.	[72,73]
	Other studies reported NK dysfunction in tumor-bearing LSL-BrafV600E/TPO-Cre mice with diminished splenocyte-mediated cytotoxicity, due to NK and CD8+ T cells. The treatment with exogenous IL-12 and anti-TGF-β partially restored, this diminished cytotoxicity.	[75]
**T cells**	In human PTC, lymphocyte density is associated with improved overall survival and lower recurrences.	[82,83]
	A study showed that proliferating lymphocytes could predict improved disease-free survival in children and young adults.	[84]
	Infiltration of CD8+ T cells into thyroid tumors was associated with improved disease-free survival. CD8+, CD4+ T cells, and B cells were positively correlated with reduced tumor sizes.	[35]
	A study found a higher risk of relapse in the presence of elevated infiltration of CD8+ T cells.	[85]
	Tregs switch off immune responses, favoring disease progression and metastases to lymph nodes in different tumors; their presence in PTCs is associated with a more aggressive disease.	[93,94]
	The percentage of Treg increased in peripheral blood and in the tumor tissues of PTC patients compared to that of MNG patients, and it was associated with aggressiveness.	[95]
	A higher density of double-negative T cells has been reported in TC patients. These T cells seem to reduce the proliferation and cytokine production of neighboring activated effector T cells.	[94,97]
**Mast cells**	MCs infiltration was reported in 95% of PTC samples whose extent correlated with extrathyroidal extension of tumors, they are also present in PDTC and ATC, and their density correlates with tumor invasiveness.	[113]
	A study revealed a higher presence in the intratumoral and peritumoral areas of follicular variant of PTC in comparison to adenoma.	[114]
	A protumorigenic role of MCs and their mediators in TC has been shown.	[112]
	MCs, by releasing specific mediators as CXCL8/IL-8, improve the acquisition of mesenchymal and stem-like characteristics of TC cells, therefore promoting cancer progression.	[113]

ATCs: Anaplastic thyroid cancers; DCs: Dendritic cells; MCs: Mast cells; NK: Natural Killer; NLR: Neutrophil-to-lymphocyte ratio; PDTC: Poorly differentiated thyroid cancer; PTC: Papillary thyroid cancer; TAMs: Tumor-associated macrophages; TANs: Tumor-associated neutrophils; TC: Thyroid cancer; TME: Tumor microenvironment.
